# Poly(ADP-ribosyl)ation by PARP1: reaction mechanism and regulatory proteins

**DOI:** 10.1093/nar/gkz120

**Published:** 2019-02-25

**Authors:** Elizaveta E Alemasova, Olga I Lavrik

**Affiliations:** 1 Institute of Chemical Biology and Fundamental Medicine, SB RAS, Novosibirsk 630090, Russia; 2 Novosibirsk State University, Novosibirsk 630090, Russia

## Abstract

Poly(ADP-ribosyl)ation (PARylation) is posttranslational modification of proteins by linear or branched chains of ADP-ribose units, originating from NAD+. The central enzyme for PAR production in cells and the main target of poly(ADP-ribosyl)ation during DNA damage is poly(ADP-ribose) polymerase 1 (PARP1). PARP1 ability to function as a catalytic and acceptor protein simultaneously made a considerable contribution to accumulation of contradictory data. This topic is directly related to other questions, such as the stoichiometry of PARP1 molecules in auto-modification reaction, direction of the chain growth during PAR elongation and functional coupling of PARP1 with PARylation targets. Besides DNA damage necessary for the folding of catalytically active PARP1, other mechanisms appear to be required for the relevant intensity and specificity of PARylation reaction. Indeed, in recent years, PARP research has been enriched by the discovery of novel PARP1 interaction partners modulating its enzymatic activity. Understanding the details of PARP1 catalytic mechanism and its regulation is especially important in light of PARP-targeted therapy and may significantly aid to PARP inhibitors drug design. In this review we summarize old and up-to-date literature to clarify several points concerning PARylation mechanism and discuss different ways for regulation of PAR synthesis by accessory proteins reported thus far.

## INTRODUCTION

Poly(ADP-ribosyl)ation (PARylation) is a special case of ADP-ribosylation—a phylogenetically ancient reaction of the transfer of ADP-ribose residues from NAD+ onto target substrates catalyzed by (ADP-ribosyl)transferases. Poly(ADP-ribosyl)ation reactions are widely used in eukaryotes, as PARP genes are absent in only a small number of eukaryotic species (1). PARP homologues apparently acquired through horizontal gene transfer can be found in bacteria (1). Interestingly, PARP from bacterium *Herpetosiphon aurantiacus* is activated by DNA like human PARP1 and can synthesize PAR polymers up to ∼15 units long (2). A protein with oligo(ADP-ribosyl)transferase activity was found in the archaeon (3). Moreover, PARP genes probably gained from their hosts were identified in a number of dsDNA viruses (1).

Among the 17-member (ADP-ribosyl)transferase protein family of mammals, only first 6 enzymes (PARP1-6) share a conserved His-Tyr-Glu (H-Y-E) triad (‘ART signature') in their catalytic domains and may be considered as ‘*bona fide*' PARPs (4). Despite this motif was predicted to be indicative for PAR-generating (ADP-ribosyl)transferases (5), the data concerning PARP3 PARylation activity are disputable (6,7), and PARP4/vaultPARP is mono(ADP-ribosyl)transferase by itself (6). PARP1, PARP2, PARP5 (PARP5a, Tankyrase1) and PARP6 (PARP5b, Tankyrase2) were shown to incorporate ADP-ribose units in a manner consistent with PAR synthesis (6,8).

Poly(ADP-ribose) (PAR), synthesized by the nuclear enzyme that was later referred to as PAR-polymerase (PARP), was first discovered in 1963 (9). Despite almost 60 years of PARP research, not all the questions concerning the mechanism of PAR synthesis have been answered to date. It is known that polymers of ADP-ribose attached to PARP1—the central cellular PAR-polymerase (10)—during auto-modification reactions may reach more than 200 residues in size and up to 100 nm in length (11,12). Moreover, PAR molecules on PARylated PARP1 adopt a highly branched ‘star' shape, as can be seen via electron microscopy (12,13) and atomic force microscopy (AFM) imaging (14). It is not evident how such a complicated structure can be achieved in unimolecular auto-modification if the polymers are elongated by ‘protein-distal' addition. However, there is a number of convincing lines of evidence that PARP1 acts as a monomer (15,16), as well as that new residues, during elongation, are attached at the PARP-distal terminus of existing PAR chains (17–19).

Several effectors of PARP1 activity, including proteins, have been discovered thus far. This includes, for example, the new regulatory proteins for PARylation system identified just recently—Sam68 (20), HPF1 (21,22) and YB-1 (23–27). Yet, in many cases, we can only speculate what the molecular mechanisms underlying their influence are on the reaction comprising PAR synthesis by PARP1. Therefore, in this review, we seek to find rational explanations for the phenomena observed.

Herein, we will consider the mechanism of poly(ADP-ribosyl)ation in detail (why DNA is necessary as a cofactor? How and where does the new ADP-ribose monomer come into play? How do individual PARP1 mutations work? What structural features endow PARP1 and limit other (ADP-ribosyl)transferases with the ability for polymer synthesis?) and attempt to determine how many PARP1 molecules are involved in auto-modification reactions. We will also try to bring together knowledge surrounding protein PARP1 regulators in order to establish how the PARP1 activity can be regulated.

## THE ‘STRUCTURAL BASIS' OF POLY(ADP-RIBOSYL)ATION

Before proceeding to a discussion of the direction of poly(ADP-ribose) synthesis by PARP1 and the data regarding whether one or two enzyme molecules are necessary for this process, we would like consider what we know about the catalytic mechanism of PARP1.

### The role of damaged DNA in PAR synthesis

**Figure 1.**
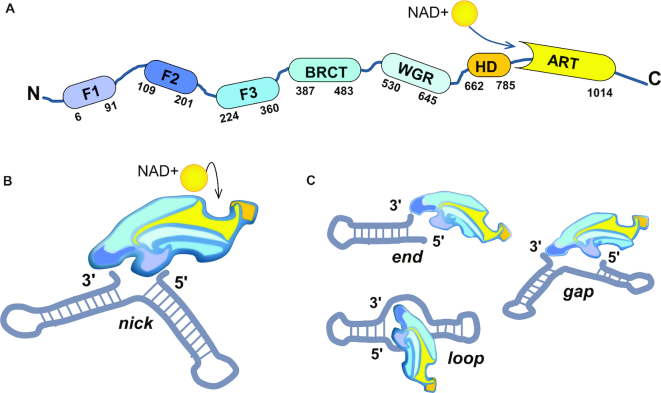
PARP1 interaction with DNA is necessary for organisation of the catalytic centre. (**A**) Domain structure of PARP1. F1-F3—zinc fingers 1–3 (F1F2 operates in DNA recognition, F3 is necessary for allosteric activation); BRCT—BRCA1 C-terminal domain (is dispensable for PARP1 activation, contains auto-modification sites); WGR—Trp-Gly-Arg domain (indispensable for the transfer of activation from F1F2 to the catalytic domain); HD—helical subdomain of the catalytic domain (auto-inhibitory); ART—(ADP-ribosyl)transferase domain (contains the active site and a fold, conserved in all PARP family members). (**B**) DNA-induced PARP1 folding. 1) F1F2 binds DNA nick *in only one orientation* (F2 on the 3′ stem, F1 on the 5′ stem (15)), directing the assembly of remaining PARP1 molecule; 2) F3 binds to the F3 binding surface created by F1 and DNA. Owing to versatile interaction between F1 and F3, a single point mutation at the interaction surface (W246A) completely abolishes activation of the full-length PARP1 (15). For the same reason, PARP1 cleavage at the F2-F3 linker by caspase 3 during apoptosis results in PARP1 inactivation (15) despite other mixtures of PARP1 fragments being able to restore the enzymatic activity; 3) WGR binds to the surface composed by DNA, F1 and F3. BRCT-WGR linker remains flexible and is able to reach the active centre of PARP1 during auto-modification of the enzyme; 4) PARP1 catalytic domain interacts with the surface organised by WGR and F3 (15), HD subdomain is unfolded, allowing productive NAD+ binding by PARP1 ART (29). (**C**) PARP1 activation by different DNA structures. Initial recognition of 3′ stem by F2 results in DNA distortions and exposure of 5′ site (15). Subsequent scanning for this site by flexibly linked F1 zinc finger permits PARP1 to effectively recognise DNA single-strand breaks with different gap lengths and double-strand breaks (15). It is possible that the recognition of other non-B DNA structures, like DNA hairpins, crosses and loops (118), can occur via an analogous mechanism.

Interaction with damaged DNA activates the reaction of poly(ADP-ribosyl)ation. The folding of the catalytically active enzyme underlies DNA-dependence of this reaction catalysed with PARP1. In the free state, this protein consists of six independent domains connected by flexible linkers like ‘beads on a string' (Figure 1A) (15). Recognition of exposed bases at the DNA damage site by PARP1 zinc fingers, F1 and F2, induces PARP1 self-assembly, with each subsequent step of the process reducing the conformational space of the system (Figure 1B,C) (15,28). The resultant decrease of entropy during inter-domain communication provides the free energy for local destabilization of the PARP1 auto-inhibitory region—the helical subdomain (HD) of the PARP1 catalytic domain (29). By using the non-hydrolysable NAD+ analogue, benzamide adenine dinucleotide (BAD), it was found that HD in its folded state entirely blocks NAD+ binding to the active site of the enzyme (30). However, in the absence of DNA, HD can transiently adopt a conformation compatible with NAD+ binding, providing the low basal activity of PARP1 (30).

Interestingly, other DNA-dependent PARPs, PARP2 and PARP3, share with PARP1 not only C-terminal regions (WGR + CAT domains), but also this allosteric regulatory mechanism of DNA-induced activation via local destabilization of HD (31). Of note, the ability of DNA-dependent PARPs to covalently modify strand break termini in DNA fragments, as discovered recently (32,33), suggests an appealing idea that the end of a DNA nick could also serve as a ‘primer' for PAR synthesis. However, it was also found that PARP-catalyzed DNA (ADP-ribosyl)ation necessitates the presence of at least two DNA strand breaks, with the first being employed for enzyme binding and activation, and the second operating as an acceptor for modification (32,33). Unlike the PARP-activating site, the acceptor site does not require the high affinity of poly(ADP-ribose)-polymerase for it, but should be free from the bound protein and placed at a well-defined distance from the PARP-activating site to be accessible for the catalytic (CAT) domain (32,33).

### The structural features of PARP1 CAT domain and its catalytic activity

**Figure 2.**
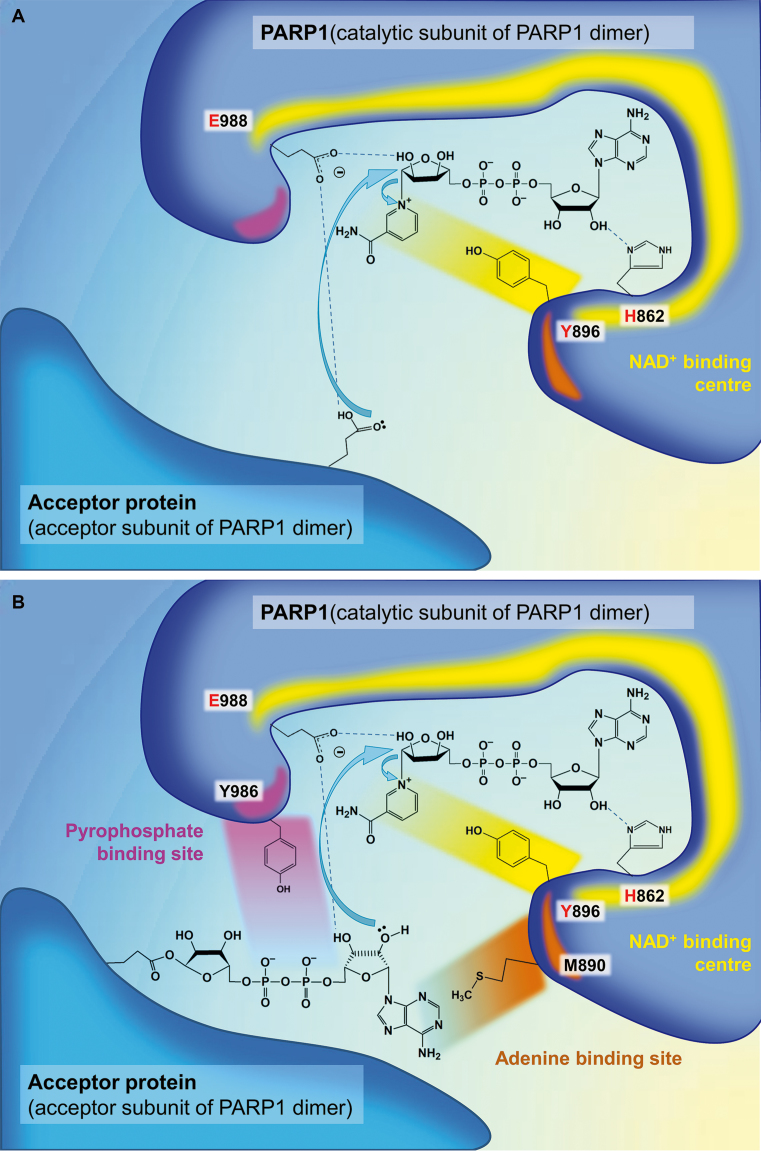

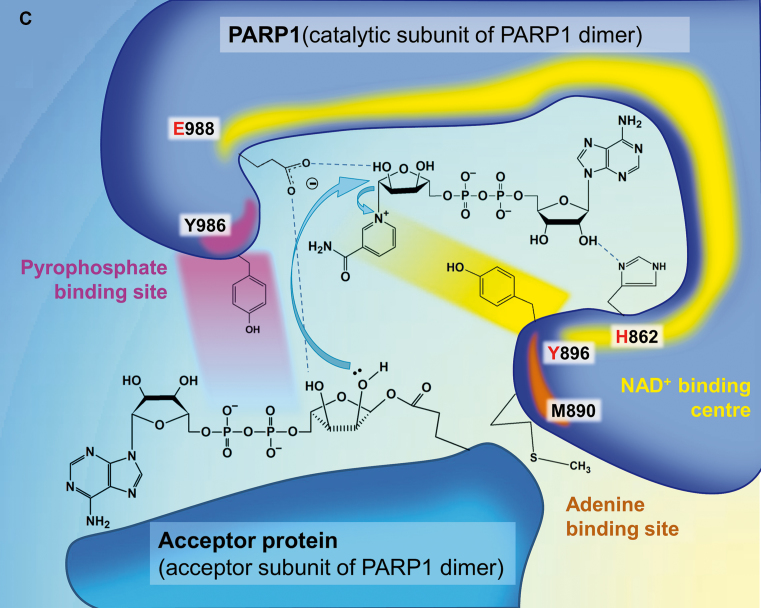
PARP1 catalytic mechanism. (**A**) Initiation. (**B**) Elongation. (**C**) Branching. The key amino acid residues of the donor site (‘H-Y-E triad': His862, Tyr896, E988) are shown in red. Important residues of the acceptor site, Met890 and Tyr986, of which mutations were especially indicative for understanding the mechanism of PAR synthesis, are also presented. Mutation M890V reduces PARP1 activity more than 200-fold (39) because of a clash between the side chain of Val890 and that of Tyr896 or the N1-atom of ADP, resulting in displacement of the accepting ribose from the PAR-binding site (37). Met890 in PARP3 acceptor site is replaced by an arginine (Arg408 in PARP3) forming a salt bridge with Asp455 (119). It is possible that this amino acid change results in decreased length of PAR polymers synthesized by PARP3 compared to PARP1 and PARP2 (36). Mutation Y986H increases the affinity of the PAR-binding site to the pyrophosphate moiety (37), resulting in 15-fold higher branching compared to wild-type PARP1 (39). Mutations E988D and E988Q reduced PARP1 elongation activity 20- and 2800-fold, correspondingly, indicating the importance of the Glu988 side chain carboxylate in this reaction (41). More information on different mutations in the PARP1 active centre and their consequences can be found in the original works (37,39,41).

The catalytic core of PARP1, (ADP-ribosyl)transferase (ART) domain, is highly conserved in all PARP family members and shares great structural similarity with the bacterial (ADP-ribosyl)ating enzymes such as the diphteria toxin (34). The ART domain is composed of a donor (NAD+-binding) site that positions the ‘donor' ADP-ribose for the transferase reaction and an acceptor site that binds either the PARylation target during initiation or the distal ADP-ribose monomer of the growing PAR chain (‘acceptor') during elongation/branching stages (35). The donor site is formed by a nicotinamide-binding pocket, a phosphate-binding site and an adenine-ribose-binding site (described in details in (36)). The nicotinamide-binding pocket contains a conserved His-Tyr-Glu (H-Y-E) triad (‘ART signature') (illustrated at the Figure 2) that is common for PARPs1-6, but is altered in other (ADP-ribosyl)-transferases. Two first amino acid residues of the triad, His862 and Tyr896, are required for binding of NAD+, while Glu988, besides substrate positioning, is also critical for catalysis. His862 binds to the 2′-OH of NAD+ adenine-ribose and its substitutions interfering with the binding of NAD+ are found in catalytically inactive PARP9 and PARP13 (6,35,36). Tyr896 stacks with the nicotinamide ring (36). Glu988 forms a hydrogen bond with the 2′-OH of the nicotinamide ribose thus polarizing the donor NAD+ molecule for nucleophilic attack (37). Natural replacement of this Glu residue by Leu, Ile, Tyr, Val or Thr observed in PARPs 6–8,10–12 and 14–16 results in elimination of PAR elongation activity, and these PARP family members act primarily as mono(ADP-ribose) transferases *in vitro* (6,35).

Apart from NAD+-coordinating amino acid residues forming PARP triad motif, secondary structural features of the catalytic domain varying within PARP family were also proposed to influence catalytic activity of the enzyme (6). Thus, the donor site loop (D-loop) shapes the donor site and interacts with NAD+ (38). The acceptor site is partly lined by acceptor loop, which structure could contribute to PARP ability to bind an incoming ADP-ribose moiety of growing PAR chain and impact elongation and branching reactions (6). Indeed, chimera PARP1 with the D-loop swapped on that from PARP16, was unable to generate PAR polymers and exhibited only mono(ADP-ribosyl)ation activity similar to PARP1 E988 mutants (see below), confirming the role of the D-loop in catalysis (6). In contrast, analogous replacement of the acceptor loop resulted only in significant reduction of NAD+ incorporation into PAR chains, revealing the importance of this structural element in elongation (6). D-loop also presents a major structural difference between catalytic domains of PARP3 and PARP1/2 (6,36). The smaller size and several amino acid changes of PARP3 D-loop compared to this region in PARP1/2 (36) were proposed to potentially account for PARP3 functioning as mono-, but not poly(ADP-ribosyl)transferase (6). Interestingly, mutations of three Pro residues of the PARP1 D-loop to Ala allowed PARP1 to generate longer PAR polymers (6).

### Mechanisms of initiation, elongation and branching

PARP1 catalytic domain is responsible for the catalysis of three chemically different enzymatic reactions during PAR synthesis: (i) the attachment of the first ADP-ribose monomer to the amino acid residue in an acceptor protein (initiation); (ii) the formation of a (2′-1″) ribose-ribose glycosidic bond (elongation) and (iii) the formation of a (2″-1′″) ribose-ribose bond between ADP-ribose units (branching) (39).

Sequence similarities between the catalytic regions of PARP1 and several ADP-ribosylating toxins (40) have allowed proposing analogies in terms of active site structure and catalytic mechanism (41). The amino acid residues playing the key roles in PAR synthesis were identified by mutagenesis (37,39,41) and crystal structures (37,42). It was put forth that Glu988 could act during initiation by facilitating nucleophilic attack by an acceptor amino acid residue in the target protein within the nicotinamide-ribose bond (41) (Figure 2A). It should be mentioned that complete loss-of-function PARP1 mutants affected with respect to initiation reactions have been never obtained (39). This confirms that acceptor residues in the PARylation target can behave as intrinsic nucleophiles not requiring activation by Glu988 during the initiation step (for instance, carboxyl groups are predominantly ionized at neutral pH) (41) (Figure 2A). On the contrary, the ability of the Glu988 carboxyl group to serve as general base activating the incoming nucleophile (2′-OH of adenosine ribose or nicotinamide ribose of the terminal PAR unit) appears to play a primary role in elongation (41) (Figure 2B) or branching as a sub-type of elongation (37) (Figure 2C). It was clearly shown that Glu988 also serves for substrate positioning. For the nucleophilic attack on NAD+, the 2′-OH of the distal ADP-ribose unit is held in place by hydrogen bonding to one of the carboxyl oxygens of Glu988 (37,41). The other carboxyl oxygen of Glu988 forms a hydrogen bond to the 3′-OH of the adenosine ribose to adjust its orientation (37) (Figure 2A, B). Although the 3′-hydroxyl doesn’t form the glycosidic linkage during PAR formation, it is required for full elongation activity, and 3′-deoxy-NAD analogues display significantly decreased substrate properties during PARylation (18,19). During the branching reaction, Glu988 seems to function in a similar manner to properly position and activate the 2″-OH of the terminal nicotinamide ribose (37) (Figure 2C).

The binding properties of the PAR-binding site have also allowed Ruf *et al.* to propose a mechanism for the branching reaction as well as explain the dual specificity of PARP1 for elongation and branching, with the first reaction being preferable (37). As was evaluated from the electron density, the pyrophosphate of ADP-ribose is tightly bound in the PAR acceptor site based on the formation of hydrogen bonds with several PARP1 residues, while the adenine is bound more weakly (37). Apart from the adenine, the ADP-ribose unit possesses internal symmetry, and the active site cleft of PARP1 is open on both sides, able to bind the PAR polymer in both orientations (37). The authors (37) posited that branching takes place when the orientation of the bound PAR is reversed by a 180^○^ rotation compared to elongation geometry (Figure 2C). The only detected PARP1 mutant, Y986H, that increases the branching:elongation ratio (from the usual 0.02 to 1.0 (39)) was suggested to have enhanced affinity of the PAR-binding site to the pyrophosphate residue (37). Stronger interactions with pyrophosphate of the PARP1 mutant may reduce the contribution of weak adenine binding in ADP-ribose positioning, thus raising the symmetry of the acceptor site (37). Enhanced probability of ADP-ribose housing in both orientations is followed by an increased branching:elongation ratio (37). It should be mentioned that other amino acid residues of PARP1 affecting branching are located at the surface of the protein molecule (39). Hence, it is possible that protein-protein interactions may influence PAR branching (39). Notably, it was determined in recent times that the level of branch chain formation is elevated by the activity of poly(ADP-ribose)-polymerase 2 (PARP2), activated by PARP1-generated PAR to catalyse additional ADP-ribosylation on top of the PAR chains (43).

### Several aspects of target recognition

The multiple amino acid acceptors of PARylation, such as Lys, Arg, Glu, Asp, Cys, Ser, Thr, pSer (phospho-serine, through the phosphate group), His and Tyr residues were proposed by proteomic approaches (44–50). It was found that the sequence and structural features that limit some ADP-ribosyl-transferases to mono(ADP-ribosyl)ation activity do not affect selectivity of amino acid targets (6). Since the NAD+ cleavage during initiation is the rate-limiting step for PAR synthesis, it was even proposed that the attachment of PAR onto acceptor proteins is advantaged by the availability of suitable amino acids, and therefore the modification of certain sites may be context-dependent (45). Indeed, solvent accessibility of Glu residues was supposed to be an important determining factor for whether a residue is (ADP-ribosyl)ated (50). Nevertheless, apparently, the position of modification sites in the target substrate is limited also by some sequence constraints. Thus, several consensus motifs (such as PXE*, E*P, PXXE* and E*XXG) were identified to surround a target Glu (E*) residues in PARylated proteins (50). Most of identified ADP-ribosylated Ser residues in modification targets are preceded by basic residues (either Lys or Arg) (21,51) that may be a key determinant for substrate recognition (52). However, to date the mechanism of target recognition by PARP1 and site-specific PARylation is elusive in large part. Intriguingly, it has been found recently that the presence of regulatory proteins may influence acceptor specificity of PARP1 (21,52).

### The direction of PAR synthesis

According to the reaction mechanism described earlier, elongation of PAR polymers by PARP1 is carried out by the addition of new ADP-ribose units to the 2′-OH terminus of the growing chain distal to the PARylation target. This model of PAR synthesis appears to be unchallenged in the case of *trans-* (or *hetero-*) modification, when active PARP1 covalently modifies another protein or the second PARP1 molecule (so-called intermolecular auto-modification of PARP1). However, considering the multiplicity of PAR acceptor sites in PARP1 (44,53–56) as well as the complex structure of the resulting polymer, it is difficult to imagine the three-dimensional geometry of intramolecular (monomeric) PARP1 auto-modification. Therefore, the alternative model of PAR synthesis as proposed by Ikejima *et al.* (57) seems to be quite reasonable in this case and should be discussed.

**Figure 3.**
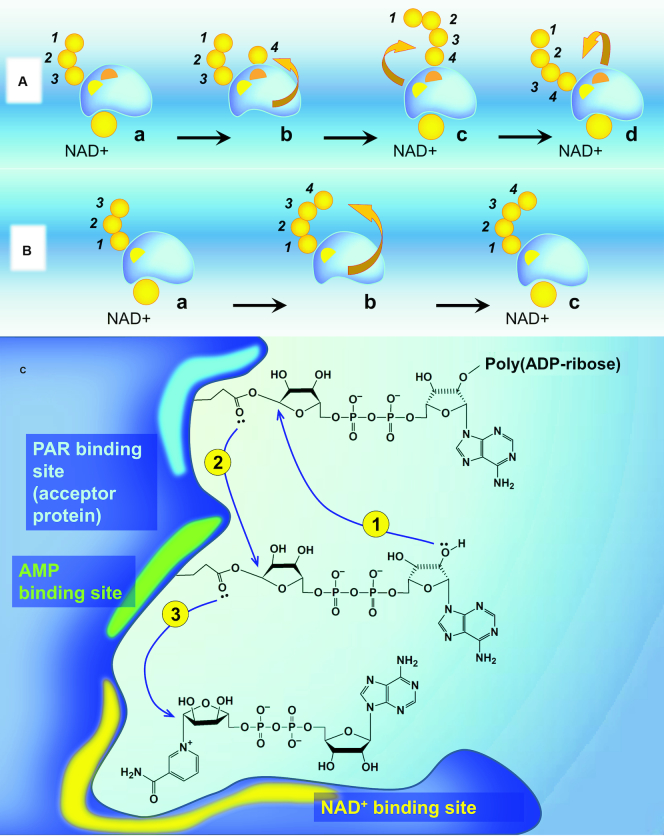
The direction of PAR synthesis. (**A**) A ‘head-out' mechanism of PAR elongation (the proximal addition model). PARP1 is first shown (a) with a partially formed PAR chain attached to the PAR binding site (yellow) and with NAD+ bound non-covalently in the NAD+ binding site. When NAD+ loses nicotinamide, its ADP-ribose occupies the AMP binding site (orange) (b). Then the PAR chain is transferred to the 2′-OH of the new ADP-ribose monomer and temporally located at the monomer site (c) before being translocated back to the PAR binding site (d) (57). (**B**) A ‘tail-out' (distal addition) mechanism. The 2′-OH of the ADP-ribose unit distal to PARP1 performs the nucleophilic attack on the NAD+ molecule held in the NAD+ binding site. (**C**) A hypothetical mechanism of PARP1-proximal elongation. (1) The transfer of the growing PAR chain to the 2′-OH of the new ADP-ribose monomer located at the monomer (AMP-binding) site; (2) Translocation of elongated PAR polymer back to the PAR binding site; (3) Occupation of AMP-binding site by NAD+ ADP-ribose (new monomer).

*A head-out mechanism* (‘proximal addition' model) (57). According to this mechanism, a poly(ADP-ribose) chain grows by addition of new ADP-ribose units at the 1″ terminus adjacent to PARP1 (Figure 3A,C). Such proximal incorporation of a new monomer necessitates at least two active centres (for ADP-ribose and PAR), which, when being used, alternately permit a chain to be elongated (similar to peptide synthesis on ribosomes). Despite this model seeming to be applicable only to auto-modification of PARP1, it is possible that a reactive intermediate at a certain stage of the elongation cycle could also be transferred to another acceptor molecule in that vicinity (57). Moreover, the same outflow of a reactive intermediate product may also account for polymer branching if another PAR chain serves as the interaction target (57). The head-out mechanism of PAR elongation was hypothesized based on the data garnered by pulse-and-chase experiments with radioactive and nonradioactive NAD+ along with experiments featuring a 2′-deoxy NAD analogue (57). However, their results were presumably misinterpreted owing to incomplete initial modification of all accessible PAR acceptor sites and PARP1 molecules because of the processivity of PAR synthesis shown later (58).

**Figure 4.**
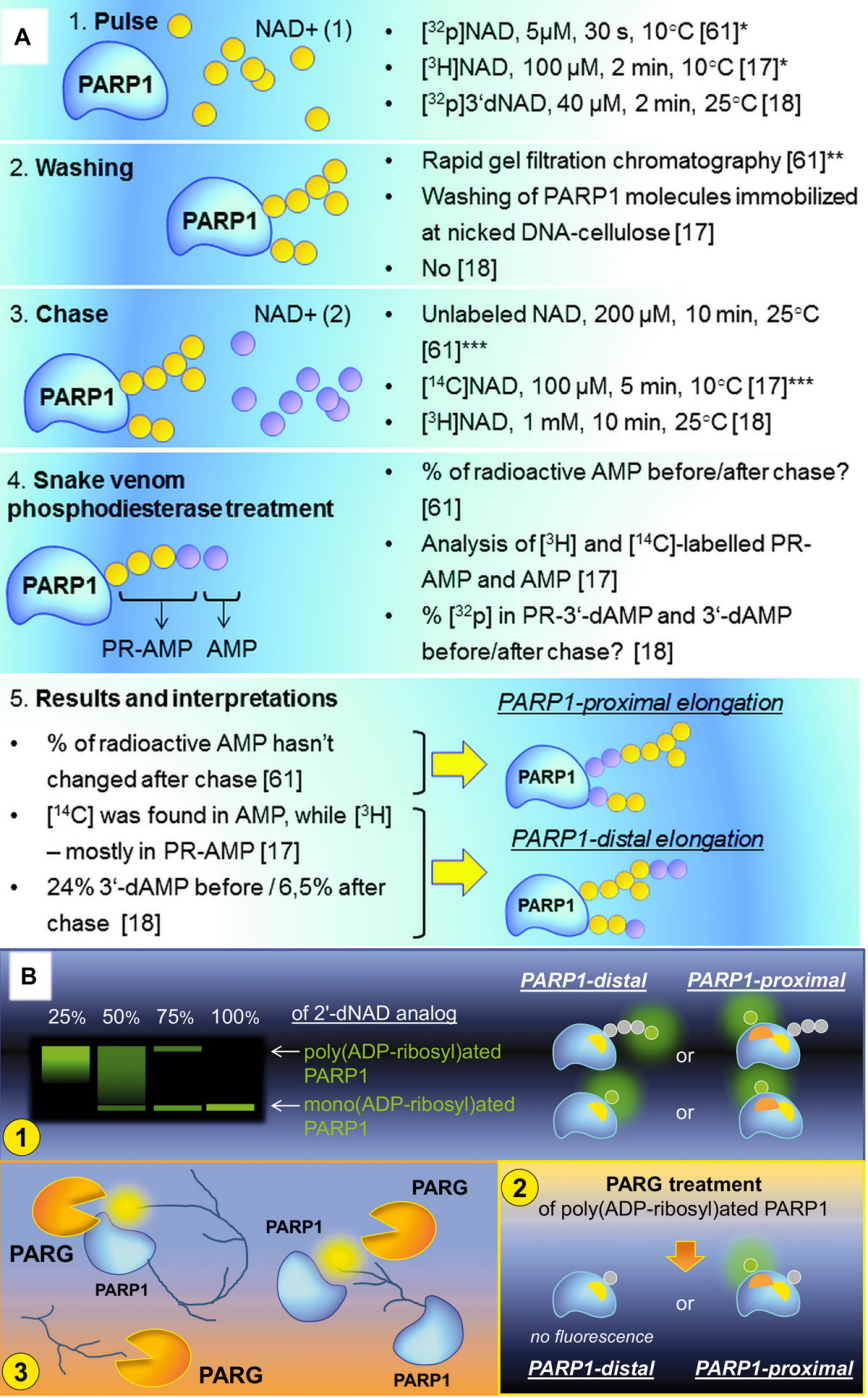
What attempts to establish the direction of PAR elongation were made? (**A**) Pulse-and-chase experiments (17,18,57). PR-AMP—phosphoribosyl-AMP. Potential problems of certain steps are indicated: (*) Not all PARP1 molecules or PAR acceptor sites were modified during the pulse stage; (**) the removal of the unincorporated NAD+ used in the pulse reaction was not full; (***) potential PAR branching at the pulse stage. (**B**) (1) PARP1 modification by ‘chain-terminating' 2′-deoxy NAD+ analogue in the presence of natural NAD+ (19). (2) We propose that additional experiments with PARG treatment would be helpful to distinguish possible situations shown in the panel (Figure 4B, (1)). PAR-binding site in PARP1 is shown with yellow, AMP-binding site is shown in orange. (3) If PARP1 elongates PAR chains according to the protein-distal model, it could compete with PARG for the distal end of the polymer by binding the terminal ADP-ribose moiety within the active site. Non-covalent interaction of PAR and PARP1 active site is shown in yellow.

*A tail-out mechanism* (‘distal addition' model). This model suggests that elongation of PAR takes place by sequential addition of the next ADP-ribose residues to the 2′-OH terminus of the chain-end ADP-ribose moiety (Figure 3B). This mechanism of polymer growth was first evidenced by pulse-and-chase experiments with NAD+ carrying different radioactive labels, [^3^H] and [^14^C] (17). This experiment similar to that mentioned previously in (57) was reported at the same time by Taniguchi (17) with the advantage that the method applied for quantitative removal of the unincorporated NAD+ was used in the pulse reaction (Figure 4A). This gave the author the opportunity to employ other reaction conditions during the pulse stage (100 μM NAD+, 2 min of incubation with PARP1 (17), compared to 5 μM NAD+, 30 s used in (57)) and therefore decrease a number of unmodified sites on PARP1 prone to PARylation in the chase reaction alone (18). The strategy was further improved by Alvarez-Gonzalez, who excogitated utilising 3′-deoxy-NAD+ in the pulse reaction (18). Owing to decreased substrate properties for PARP1, especially in the elongation reaction (18), this NAD+ analogue turned out to be an ideal variant for modification of the majority of PAR acceptor sites by short (1–4 units) ADP-ribose oligomers at the pulse stage (18).

By now, the limitation of PAR chain growth by the addition of 2′-deoxy NAD+ analogues can be elegantly detected by click chemistry with fluorescent dyes (19). Despite this technique allowing observing incorporation of ‘chain-terminating' NAD+ analogues in PARP1 modified by long PAR polymers, the Figures provided in (19) alone cannot fully disprove the ‘proximal addition' model (Figure 4B, 1) and ideally should be supplemented with experiments featuring poly(ADP-ribose)-glycohydrolase (PARG) treatment, that would clearly demonstrate disappearance of fluorescent signal based on removal of the modified ADP-ribose units incorporated in the distal ends of PAR chains (illustrated in Figure 4B, 2).

Therefore, today, the ‘distal addition' model of PAR synthesis can be considered established, especially in light of identification of PARP1 regions involved in poly(ADP-ribosyl)ation with no evidence for the existence of two ‘switching over' active centres. The mechanism of PARylation discussed previously also illustrates the chemical difference between the initiation and elongation steps. This corresponds to the finding that PARP1 catalyses initiation in a distributive and elongation in a processive manner, respectively, with the elongation rate exceeding that of initiation 232 times (58–60). Contrastingly, we could more likely expect equality of these two steps in the case of the protein-proximal model owing to re-attachment of the growing PAR to the acceptor in every cycle (Figure 3C). An interesting confirmation of the tail-out mechanism is also the finding that the rate of degradation of protein-bound PAR is not proportional to the concentration of PARG: a 4-fold increase in PARG gives a 2-fold increase in degradation rate, while free PAR chains are degraded at a rate directly proportional to the PARG concentration (61). This result suggests that there is direct competition between PARP1 and PARG for the polymer (61) (illustrated in Figure 4B, 3). PARG acts predominantly as an exo-glycohydrolase, binding the PAR terminus distal to the acceptor protein and is unable to release the most proximal ADP-ribose residue (2).

To conclude, the direction of PAR elongation appears to be protein-distal, though there is no absolutely incontestable evidence for this.

## THE STOICHIOMETRY OF PARP1 POLYPEPTIDES IN THE REACTION OF AUTO-PARYLATION

Usually, the question is framed like this: does auto-modification of PARP1 occur in a dimeric (requiring two molecules of the enzyme) or monomeric mode (one protein molecule is sufficient)?

**Figure 5.**
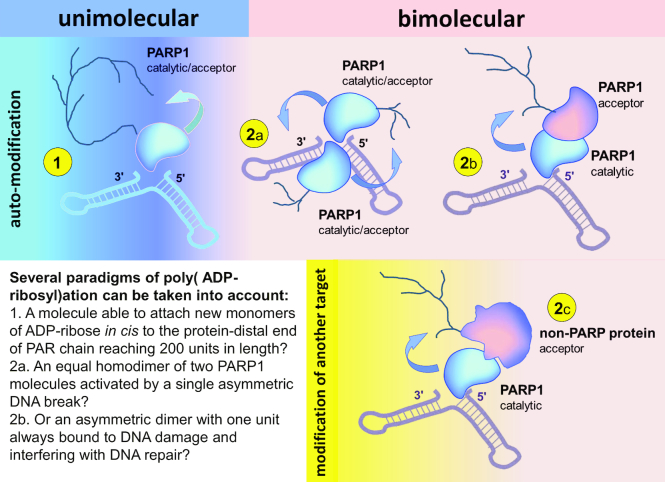
Poly(ADP-ribosyl)ation in dimers and monomers: terminology and different paradigms. (1) Unimolecular (*mono*molecular, *intra*molecular, *in cis* modification). One molecule of PARP1 interacts with DNA break, becomes catalytically active and modifies itself (*as a monomer*). In this case one PARP1 polypeptide serves as a catalyst and an acceptor of poly(ADP-ribosyl)ation at the same time. (2) Bimolecular (*inter*molecular, *hetero-* or *in trans* modification) occurs in protein dimers: (2a) ‘*Homodimers*' of PARP1. The dimer of two PARP1 molecules is formed by protein-protein interactions. Binding of the first molecule to the DNA break induces its interdomain rearrangement, resulting in both activation of this molecule and symmetric self-assembly of the second PARP1 molecule driven by rearrangement of the protein-protein interaction surface. The active PARP1 homodimer consists of two identical subunits, both functioning as a catalyst and acceptor of PARylation simultaneously. (2b) ‘*Heterodimers*' (‘asymmetric homodimers') of two PARP1 molecules. DNA-bound PARP1 subunit is active and functions only as a catalyst. The second PARP1 molecule is inactive and functions only as an acceptor of poly(ADP-ribose). **(2c)** ‘*Heterodimers*' of PARP1 and other proteins. DNA-bound PARP1 molecule acts as a catalyst. Another (non-PARP1) protein is a target for poly(ADP-ribosyl)ation by PARP1.

Therefore, despite the abundance of data speaking in favour of each of the paradigms, researchers still adhere to opposing views on this matter. As can be seen in Figure 5, neither of these paradigms is without contradiction with at least some of the data accumulated after almost 60 years of PARP research.

Therefore, the following questions can be asked:

1. Can one PARP1 polypeptide modify itself, acting both as a catalyst and an acceptor simultaneously?
2. Can one PARP1 molecule (catalyst) modify another PARP molecule (acceptor) just like any other target protein?
3. If the answer to the previous questions is yes, which of these processes is more important?

### PARP1 as a dimer

The supposition that PARP1 auto-modification takes place *inter*molecularly within a dimer of two PARP1 polypeptides seems especially rational taking into account the protein-distal direction of PAR chain growth and PARP1’s ability to covalently modify other proteins. Self-association of PARP1 in solution with the formation of PARP1 dimers was observed by gel-permeation chromatography and confirmed by electrophoretic separation of self-associated molecules in non-denaturing and denaturing gels as well as cross-linking experiments (62). Moreover, the authors found that glutaraldehyde-cross-linked PARP1 dimers possessed significantly increased enzymatic activity compared to monomeric PARP1 molecules (62). PARP1 dimerization was also demonstrated by dynamic light-scattering technique (63). It was also shown that PARP1 activity depends on self-association of the enzyme and is maximal for dimeric PARP1, while further multimerization and, conversely, dissociation to single PARP1 molecules leads to significantly decreased enzymatic activity (62). Similar bell-shaped dependence of the PARP1 auto-modification rate on enzyme concentration, indicating the catalytic role of PARP1 dimerization, was observed by others (59). Furthermore, in several works, it was demonstrated that the initial rate of auto-poly(ADP-ribosyl)ation increases with second-order kinetics as a function of PARP1 / PARP1 catalytic domain concentration (59,60,64) and NAD+ concentration at low levels (59,65). These findings suggest that two molecules of PARP1, each carrying one NAD+ molecule in its single NAD-binding site (66), are required for auto-modification (59).

Additional compelling evidence for the bimolecular nature of the auto-poly(ADP-ribosyl)ation reaction is PARP1:DNA binding stoichiometry, which appears to be 2:1 for optimal enzyme activity (67–69). Interestingly, the second protein molecule was found to bind the PARP1-activating site in DNA with a higher affinity than the first molecule, indicating cooperative binding (67). Resulting high-affinity interactions of PARP1 dimers with DNA can be confirmed by footprinting experiments and correlate with maximal PARP1 activity (67). On the contrary, when binding constants for the first and second PARP1 molecules are equal, no protection of DNA is observed by footprinting data, and PARP1 activity is low (67).

The functional role of PARP1 dimerization is also validated by crystallography data. The formation of a homodimer interface between two F3 monomers visible from the crystal structure of the PARP1 F3 zinc finger domain suggests that the F3 monomers may exist as a dimer in the DNA-activated state of PARP1 (70). The crystal structure of the two first PARP1 zinc-finger domains, F1 and F2, bound to DNA breakage, reveals dimerization of PARP1, with F1 and F2 zinc fingers from separate protein molecules forming a recognition module (68). Uniquely, the distance between the F1 C-terminus and F2 N-terminus observed in crystals was found to be too extensive to be spanned by the linker separating these domains within a single PARP1 polypeptide without serious steric clashes, inferring intermolecular interaction of two PARP1 molecules during DNA recognition (68).

As a matter of fact, the best evidence for PARP1 dimerization is the PARP1 ability to function as the enzyme consisting of several independent modules that when added separately, can create a fully functional enzyme. Thus, the mixture of individual PARP1 domains in different combinations is able to restore PARylation activity comparable to that intrinsic to the wild-type enzyme (ΔF1 + F1 or ΔF1 + F1-F2 (68), ΔF2 + F2 or ΔF2 + F1-F2 (68), ΔF3 + F3 (71), F1-F3 + WGR-CAT (44)). The composition of various inactive PARP1 mutants, at least *in vitro*, also results in active PARP1 (W318R + E988A (16,71), W318R + ΔWGR-CAT (16), ΔF1 + ΔF3, ΔF1 + E988K or M890V/D899N, ΔF3 + E988K or M890V/D899N, ΔF1 + ΔWGR, ΔF3 + ΔWGR (44)). Modification of inactive PARP1 mutants by wild-type PARP1 molecules, clearly indicating *in trans* PARylation, can be observed in experiments with N-terminal SUMO-tagged (SMT) variants of PARP1 (distinguishable on SDS-PAGE) (15) as well as with acrylodan-labeled PARP1 mutant, W318R, and unlabeled wtPARP1 (by a blue shift in the acrylodan-labeled W318R emission peak, indicative of a HD destabilization) (16).

To conclude:

- PARP1 is able to modify another PARP1 molecule *in trans*.
- The data on cooperative binding together with footprinting experiments speak in favour of asymmetric PARP1 dimers, in which two PARP1 molecules are unequal.
- As what follows from a large variety of active PARP1 mutant pairs, there is no unified mode for folding of active PARP1 dimers at the PARP1-activating site in DNA.

### PARP1 as a monomer

Despite much data in favour of the bimolecular nature of PARP1 auto-modification, there are convincing arguments for PARP1’s capacity to modify itself *intra*molecularly. In contrast to (70), through sedimentation equilibrium data and gel filtration analysis, it was shown that the isolated zinc finger, F3, exists as a monomer in solution (71). NMR evaluation also reports a monomeric structure of the F3 domain (72). Moreover, mutations in the F3 dimer interface incompatible with dimerization had no influence on PARP1 activity (71). Static light-scattering measurements as well as sedimentation velocity and equilibrium experiments determined that the PARP1(1–486) (F1–F3 + BRCT domains) is an elongated monomeric particle in solution (73). Sedimentation analysis indicates that full-length PARP1 also exists as a monomer in solution, and that the arrangement of PARP1 domains upon binding to DNA occurs with a 1:1 stoichiometry (74).

The monomeric mode of PARP1 binding to DNA was also exhibited by Lilyestrom with co-authors: according to electrophoretic mobility shift analysis, PARP1 (1–486) forms 1:1 complexes with 30-mer DNA-duplexes irrespective of nicks, extensions or blunt ends (73). A 1:1 binding stoichiometry was also demonstrated for PARP1 and nucleosomes (75). Binding of the F2 domain alone or the F1 + F2 PARP1 fragment to a dumbbell-nicked or gapped DNA structure was also seen to occur as a monomer, as was shown by various methods (28). Moreover, unlike in (67), the binding constants quantified by Eustermann and colleagues (28) show that in the complex of F1 + F2 PARP1 fragments with nicked dumbbell DNA, there is a single site featuring a high-affinity interaction (*K*_D_ between 5.7 nM at 0 mM NaCl and 45 nM at 200 mM NaCl), leading to a 1:1 complex, and secondary sites with significantly lower affinity (*K*_D_ varying from 0.2 μM at 0 mM NaCl to 2.6 μM at 100 mM NaCl, too weak to be quantified at greater ionic strengths). This tighter binding site corresponds to protein interactions with single-strand breaks as was eliminated in the control experiment with ligated DNA (28). It should be mentioned that DNase I footprint on nicked DNA of the F1 + F2 PARP1 fragment is identical to that of full-length PARP1 (76). Of note, despite these data clearly indicating the monomeric mode of PARP1 interaction with respect to the PARP1-activating site in DNA, it still cannot exclude the possibility of the formation of PARP1 ‘homodimers', where the first subunit is activated by DNA binding and the second adopts a catalytically active conformation owing to rearrangements of the protein-protein interface. However, this variant is disproved by further data from the group (15) as described subsequently, because it is unlikely that a single mutation in the PARP1 catalytic centre (E988K) could significantly distort the E988K-wtPARP1 interaction surface.

AFM imaging analysis performed by Liu *et al.* also indicates that PARP1 is monomeric in solution and most PARP1 molecules remain monomeric upon binding to DNA at nicks, abasic sites and ends (77). No co-localization of the two colors, corresponding to dimerization of PARP1 molecules labeled with Qdots of two different emission wavelengths, was observed in single-molecule DNA tightrope experiments (77).

Evidence that PARP1 is able to modify itself intramolecularly may be obtained from the experiments with inactive mutant forms of PARP1. Hence, both W318R and E988A mutants are shifted in the reaction when mixed, indicating that the active catalytic domain of W318R can also modify itself *in cis* (71). According to the *in vitro* activity assays of Eustermann *et al.*, PARP1 auto-modification may take place *in trans* only in the case of two closely adjacent PARP1 binding sites in DNA (e.g. opposite ends of a DNA duplex), while in the situation of single recognition site (DNA break in nicked DNA dumbbell), auto-modification occurs predominantly *in cis* (15). For this experiment, active wild-type and inactive E988K versions of normal and N-terminal SMT variants of PARP1 (distinguishable on SDS-PAGE) were mixed in the presence of different DNA structures (15). Whereas both active and inactive PARP1 molecules were modified in the presence of DNA duplexes, unequivocally indicating *trans*-modification of E988K by wild-type PARP1, just active PARP1 was shifted in the presence of nicked DNA dumbbell, providing evidence for exclusive *in cis* modification (15). Ineffective *trans*-modification of inactive mutants was observed in the presence of a hairpin DNA structure with a single DNA end (15). Later, this model was used to manifest that intramolecular PARP1 activation is biochemically preferable to intermolecular activation (16).

As the best confirmation of dimeric nature of PARP1 auto-modification is that mixing of different PARP1 mutants can result in intermolecular complementation *in vitro*, the best rebuttal for this in ‘monomeric' conception is that intermolecular PARP1 activation appears not to be a relevant mechanism in cells (16). Despite W318R and E988A mutants having demonstrated auto-modification activity when mixed together *in vitro*, cells expressing both of the mutant proteins were not able to produce detectable PAR in response to H_2_O_2_ treatment, indicating that intermolecular complementation does not take place within a cellular context (16). Nevertheless, it should be considered that in theory, mixing of W318R and E988A mutants should result in formation of only 25% of active heterodimers—those where catalytically active W318R mutants provide their WGR and CAT domains, and E988A supplies its DNA-binding platform able to transfer the DNA damage-induced activation further to the NAD-binding centre. Another conceivable reason for significantly diminished PAR production in cells expressing only PARP1 mutants compared to those expressing wild-type versions of PARP1 fusions with fluorescent labels (16) may be their capacity to form heteromeric complexes with, but inability to modify other (non-PARP1), PARylation targets.

To conclude:

- Only one PARP1 molecule is activated by a single PARP1-activating site in DNA.
- PARP1 is able to modify itself *in cis*.
- The variant of symmetric PARP1 ‘homodimer' functioning at a single DNA break appears to be disproved by the data (15).

### An asymmetric dimer of two PARP1 molecules?

Despite the data described earlier being seemingly contradictory, several speculations concerning this issue could be made:

- PARP1 auto-poly(ADP-ribosyl)ation as an intermolecular modification within a PARP1 dimer, where both subunits are equal and function as catalysts and acceptors simultaneously, is possible in the case of two closely adjacent PARP1 binding sites in DNA, but appears to be doubtful as a general mechanism.
- Invariably, only one PARP1 polypeptide can interact with a single PARP1-activating site in DNA to adopt the conformation compatible with NAD+ binding and become catalytically active.
- Therefore, in the case of a single PARP1-activating site present in DNA, intermolecular PARP1 auto-modification may occur within ‘heterodimers' of PARP1 constituted by one catalyst and one acceptor molecule. This model, repeatedly mentioned in the literature (18,59,69), is, among others, supported by the fact that initial rates of PARP1 auto-poly(ADP-ribosyl)ation at high NAD+ concentrations (10–200 μM) follow substrate saturation kinetics rather than increasing with the square of the NAD+ concentration (59). Taking into account that PARP1 can poly(ADP-ribosyl)ate other proteins, abundantly present in the nucleus and bound to chromatin (78), predisposed to act as PAR acceptors, it is safe to assume that *trans*-modification of inactive ‘acceptor' PARP1 by active DNA-bound enzyme is one of the existing means of PARP1 auto-modification. Moreover, it is possible that this mechanism is responsible for the formation of long and branched PAR chains along with multiple modification of a single PARP1 molecule because it is free from the limitations connected with the protein-distal direction of PAR chain growth and enzyme inactivation due to conformational changes (16). However, the significance of its contribution to overall auto-poly(ADP-ribosyl)ation of PARP1 remains unclear.
- If *trans*-modification within ‘heterodimers' of two PARP1 molecules with unequal functions was the only mechanism of PARP1 auto-modification, the catalyst subunit would remain unmodified and bound with the DNA damage site, interfering with the action of DNA repair systems. Actually, PARP1 in the absence of NAD+ inhibits several base excision repair enzymes (79). For this reason, PARP1 inhibitors, like olaparib, trapping PARP1 on the lesion owing to inactivation of its catalytic activity (80) as well as reversing allostery (30,81), are approved for use in the treatment of cancers with defective homologous recombination mechanisms (82). Therefore, PARP1 dissociation from complexes with damaged DNA during PARylation is indispensable for normal DNA repair; anyhow, it is well-documented. For example, DNA release may be demonstrated indirectly by reversion of DNA-induced HD destabilization of acrylodan-labeled PARP1 (16), or by decrease in fluorescence anisotropy of fluorescently labeled DNA bound by PARP1 (83), following NAD+ addition. However, in both scenarios, the authors used DNA structures predisposed to *trans-*modification of PARP1—a hairpin with a single DNA end (15,16) and DNA duplexes (83), correspondingly. There is also the potential that the unmodified catalyst subunit of PARP1 dimers can dissociate from the complex with damaged DNA as a result of non-covalent interactions with PAR, synthesized during *trans*-modification of other proteins. Nevertheless, our experiments on PARylation in the presence of PAR did not feature any influence of PAR on PARP1 activity stimulated by damaged DNA, indicating the existence of PARP1-DNA complexes under these conditions (26).
- PARP1 is able to modify itself intramolecularly as a monomer (15). According to the structural model of PARP1 bound to DNA nicks and hydrogen/deuterium exchange-mass spectrometry data, the BRCT-WGR linker remains flexible during PARP1 interaction with DNA nicks and can actually reach the active site of PARP1 to be modified *in cis* (15,29). Based on the protein-distal direction of PAR elongation, it may be proposed that this manner of auto-modification is not appropriate for the formation of long PAR chains if the polymer does not form a loop during its synthesis. However, even PARP1 auto-modification by several residues of ADP-ribose seems to be adequate for its dissociation from the damage site. Interestingly, as early as in 1987, Ikejima *et al.* (57) noted that the covalent modification by large polymer is unnecessary for the regulation of protein function because the attachment of a single ADP-ribose unit to the substrate proteins by (ADP-ribosyl)ating bacterial toxins is sufficient for dramatic changes in their activities. They suggested that massive PAR synthesis is critical for the local and temporal reorganization of nucleoprotein structures around the DNA break (57). Indeed, poly(ADP-ribose) has been recently discovered to seed for liquid demixing events that lead to the formation of a non-membranous DNA repair compartment at a DNA damage site (84).

## REGULATION OF PARP1 ACTIVITY

The question of whether PARP1 functions as a dimer or monomer is directly relevant to the issue of the regulation of PARP1 activity.

As follows from the previous section, auto-modification of PARP1 can occur either via *in trans* bimolecular modification within the PARP1 dimer, or *in cis* modification performed by a single PARP1 molecule bound to the PARP1-activating site in DNA. It should be underscored that the implementation of the second scenario results in dissociation of the enzyme from the complex with damaged DNA followed by its inactivation (16).

This means that the presence of another protein (replacing the acceptor subunit in the case of the PARP1 dimer) can interfere with both variants of enzyme auto-modification and significantly influence the overall poly(ADP-ribosyl)ation yield. Moreover, we can expect that PARylation targets and proteins not prone to PARylation would influence PARP1 activity in varying ways.

### PARP1 regulation by protein partners and posttranslational modifications

**Figure 6.**
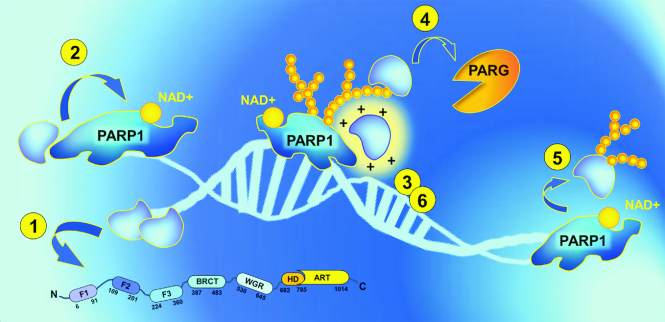
PARP1 regulation by other proteins (ideas for possible mechanisms). (**1**) *DNA-binding proteins*: could inhibit PARP1 activity based on its displacement from the DNA or stimulate the enzyme by prevention of catalytically ineffective PARP1 binding with single-stranded DNA / by modulation of DNA conformation (DNA melting). (**2**) *Proteins that physically interact with PARP1* may influence PARP1 allosteric activation. (**3**) Basic *PAR-binding proteins*: can screen the negative charge of growing PAR chains, stabilizing catalytically active PARP1-DNA complexes during PAR elongation. (**4**) *PAR-binding proteins* are also able to protect PAR from degradation by PARG and increase the life span of poly(ADP-ribose). (**5**) *PARylation targets*: could permit PARP1 to remain longer in the active non-PARylated state by providing another (non-PARP1) platform for modification. (**6**) *PAR-binding proteins* may facilitate PARylation reactions by inducing molecular crowding and raising the effective concentrations of the reaction participants.

PARP1 activity is regulated by physical interactions with other proteins (histones (85), HPF1 (22), mH2A1.1. (86), HMGN1 (87), XPA (88), NEIL1 (89), OGG1 (90), DDB2 (91), p53 (92), ERK2 (93), Sam68 (20), YB-1 (24,26), C12orf48 (94), etc.). In certain cases, these protein-protein contacts are direct and not mediated by DNA (as they remain upon DNAse I or EtBr treatment (89–91)) or PAR (they are not interrupted in cells by the presence of PARP1 inhibitors (20)). However, the interactions within PARP1 heterodimers can be strengthened quite pronouncedly in the presence of DNA (89,90) or PAR (95) owing to engaging of additional protein domains (e.g. DNA-binding domain of PARP1 (89,90)). In general, PARP1 forms protein complexes with PARylation targets, and interaction of the active enzyme and acceptor results in poly(ADP-ribosyl)ation of the target (23,85,91,95,96). It is possible to assume that stimulation of PARP1 activity usually observed in this case is related to availability of the non-PARP1 acceptor of PARylation, allowing PARP1 itself to remain longer in the active non-PARylated state (Figure 6). Quite the opposite, several PARylation targets have been shown to stimulate auto-modification of PARP1 (26,87). Sometimes, PARP1 interacts with proteins that are not prone to poly(ADP-ribosyl)ation and only serve as regulators of PARP1 enzymatic activity (22,86,88–90). In this case, different variants of PARP1 regulation are feasible: inhibition (86); stimulation (88–90); and inhibition of PARP1 auto-modification associated with stimulation and increased acceptor specificity of PARP1 *trans*-modification activity (21,22). However, the molecular mechanisms underlying these phenomena are not fully understood.

There is the potential that physical interactions of PARP1 with other proteins may affect its activity by means of alterations of PARP1 structure and/or interdomain communications that are of key importance for DNA-dependent activation of the enzyme. Posttranslational modifications of PARP1, like mono(ADP-ribosyl)ation (97,98), phosphorylation (99–101), methylation (102) and acetylation (99), can actually modulate its activity. Interestingly, PARP1 acetylation sites map to domain-interaction surfaces, including that of F1, F2 and WGR (15).

PARP1 is methylated at K508 by SET7/9 *in vitro* and *in vivo*, and this posttranslational modification significantly stimulates its enzymatic activity (102). Methylation site lies within the central auto-modification domain of PARP1, and its modification may stabilize PARP1 auto-modification domain in the catalytic cleft, thus sensitizing the enzyme for auto-modification (102). Otherwise, methylation could influence PARP1 structural rearrangements, which affect the interaction of the DNA-binding domain to the CAT domain (102).

Of special interest is PARP1 regulation by ERK2 kinase, which is able to stimulate PARP1 activity in two different manners—either by phosphorylation of PARP1 (101), or, irrespective of its kinase activity, by physical interaction with PARP1 (for this, the phosphorylated form of ERK2 itself, pERK2, is required) (93). Notably, PARP1 activation by pERK2 may occur via a DNA-independent mechanism that appears to differ from DNA-dependent PARP1 activation (93). PARP1, activated by pERK2, has higher affinity for NAD+ compared with PARP1 activated by DNA damage (93). It cannot be ruled out that pERK2 binding in this situation leads to immediate structural adjustments of the PARP1 catalytic domain without involvement of other protein regions.

An important distinctive feature of proteins found to regulate PARP1 activity is that the majority of them are PAR-binding proteins that have a high affinity for poly(ADP-ribose) (25,88,95,103). In this regard, an amazingly elegant mechanism of PAR-directed poly(ADP-ribosyl)ation has been recently proposed for p53 and potentially other proteins able to bind PAR owing to the presence of intrinsically disordered basic stretches in their structures (95). It was found that the multifunctional C-terminal domain (CTD) of p53, which is highly basic and intrinsically disordered, serves in very specific non-covalent interactions with autoPARylated PARP1 via PAR, resulting in covalent modification of p53 (95). Strikingly, the authors demonstrated that fusing the CTD of p53 to a protein that is normally not a substrate for covalent modification by PARP1 (GST) renders this protein a target for PARylation (95). A bioinformatics analysis revealed that CTD-like elements are markedly enriched in PARylated proteins, and these regions may reach 40–50 or more amino acid residues in length (95). Moreover, the charge density influences the extent of the resulting PARylation and might be elevated by multimerization of the protein in the case of a short CTD-like region, similar to p53 (95). Of interest, while electrostatic interactions of CTD with PAR allow the protein to slide along the PAR polymer towards the active center of PARP1, other structure-specific PAR-binding modules (for example, macrodomain that recognises the terminal ADP-ribose moiety of PAR) appear not to confer sufficient spatial proximity for covalent modification (95). The combination of intrinsic disorder and high positive charge was proposed to provide non-covalent interactions with PAR and subsequent covalent modification of the proteins containing CTD-like regions (95).

It is worth commenting that p53 is also able to stimulate PARP1 activity by 1.5–2 times, but has no influence on the kinetics of PAR synthesis, namely, average PAR polymer length and branching frequency (92). Therefore, it seems sensible to assume that non-covalent interaction of p53 with PAR may contribute to electrostatic stabilization of the active PARP1-DNA complex during auto-modification of the enzyme. Screening of the negative charge of growing PAR chains during elongation actually plays a major role in poly(ADP-ribosyl)ation. Unlike proteins, nucleic acids are highly charged, and bridging in close proximity of two strongly charged anions, such as two DNA molecules, much less DNA and PAR, requires overcoming an enormous electrostatic energy barrier (104). In the absence of cations, the damaged DNA itself is not an effective PARP1 cofactor (85). DNA-dependent PAR synthesis is significantly stimulated by bivalent metal ions (Mg^2+^, Ca^2+^ (85)) or multivalent cations (polyamines (85) and basic peptides). This is the explanation for why the majority of proteins able to stimulate PARP1 activity have high isoelectric points (histone H1 (105) (p*I* = 10.84), HMGN1 (87) (p*I* = 9.6), DDB2 (91) (p*I* = 9.56), YB-1 (26) (p*I* = 9.87)) or are at least PAR-binding (like XPA (88)). Of interest, acetylation of Lys residues in histones, H1 and H3, makes them ineffective PARP1 activators (85).

### Novel PARP1 regulatory proteins

#### Sam68

Src-associated substrate during mitosis of 68 kDa (Sam68) is an RNA-binding protein also able to bind single- and double-stranded DNA (20). In 2016 this protein was identified as a novel regulator of DNA lesion-triggered PAR production (20). Sam68-deleted cells and animals have impaired PAR synthesis and are hypersensitive to genotoxic stress (20). Interestingly, Sam68 deletion has a similar effect on DNA repair as PARP1 deficiency or inhibition (20). Sam68 localizes at DNA lesions independently of PARP1. Treatment of cells with PARP inhibitor PJ-34 had no influence on Sam68-PARP1 interaction, suggesting that Sam68 recruitment to the DNA damage sites is PAR-independent (20). In contrast, the interaction of two proteins greatly decreased in the presence of EtBr, indicating that damaged DNA is critical for this interaction (20). Sam68 significantly stimulates PARP1 activity in the presence of damaged DNA (without DNA detectable PARP1 activation was not observed) (20). The N-terminal regions of Sam68 (1–102 aa residues) and PARP1 (1–662 aa residues) are absolutely necessary for Sam68–PARP1 interaction and stimulation of PAR production by PARP1 (20). Of note, Sam68 does not associate with other PARPs that greatly differ from PARP1 in their N-terminal domains: PARP2, PARP3 and PARP5a/b (20). Certainly, PARP1 catalytic domain (663–1014 aa residues) is also indispensable for PAR synthesis even in the presence of Sam68 (20). Therefore, Sam68 appears to stimulate PARP1 catalytic activity via regulation of the DNA-dependent activation of enzyme (20).

#### HPF1

Another compelling example of PARP regulator is uncharacterized human protein C4orf27, also identified in 2016 and referred as histone PARylation factor 1 (HPF1). HPF1 is recruited to DNA damage sites in a PARP1-dependent manner, independently of PARP1 enzymatic activity, as loss of PARP1, but not pretreatment of cells with PARP inhibitors prevents HPF1 localization at DNA lesions (22). The C-terminal region (242–346 aa residues) of HPF1 and PARP1 CAT domain are responsible for direct physical interaction between these proteins (22). It should be mentioned that HPF1 also interacts with PARP2, but not PARP3, whose catalytic domain differs from PARP1/2 CAT domain (21). HPF1 promotes ADP-ribosylation of histones by PARP1, but limits auto-modification of the enzyme (22). Moreover, in the presence of HPF1 both *auto*- and *trans*-ADP-ribosylation activity of PARP1 becomes Ser-specific (21). The same effect of HPF1 on ADP-ribosylation reaction was observed for PARP2, but not PARP3 (21).

Two highly conserved amino acid residues in the C-terminal region of HPF1, Tyr238 and Arg239, were found to play an important role in HPF1-PARP1 interaction, as HPF1 mutants in which any of these residues are replaced by alanine are not able to bind PARP1 (22). Although Y238A/R239A mutant does not possess any notable differences in folding compared to wtHPF1 (by circular dichroism data), Y238A/R239A mutant has no influence on PARP1 activity (22). Interestingly, Tyr238 has been recently identified as a target of PARP1-catalyzed ADP-ribosylation *in vitro* and *in vivo* (48,106). The authors proposed that not Tyr itself, but namely its modification is significant for regulation of HPF1 cofactor functions during PARylation process.

Interestingly, according to the results of phylogenetic analysis, HPF1 and PARP1 might have closely coevolved, suggesting a conserved mechanism of PARP1 regulation in PARP1-containing organisms (22).

Potential mechanisms of how HPF1 switches PARP1 target specificity to Ser residues were proposed by Leung (52). Based on the HPF1 ability to interact with catalytic domain and regulate target specificity of PARP1 and 2, but not PARP3 (21,22), he hypothesized that HPF1 may possibly alter the conformation of the D-loop (52). This supposition could also explain variation in length specificity, as contrary to the well-known PARylation activity of PARP1, Ser residues in target proteins appear to be mono(ADP-ribosyl)ated (21,52). (Indeed, PARP4 provides an example of how the interaction with partner proteins may influence length specificity (52). Being a mono(ADP-ribosyl)-transferase by itself (*in vitro*), PARP4 possesses PARylation activity in its native protein-complex form (6)). Inducing conformational changes at the PARP1 catalytic centre, HPF1 binding may lead to positioning of target substrate in a way promoting presentation of its Ser residues in more accessible and/or activated state, while the otherwise modified sites of the substrate protein turn out in a less advantageous conformation (52). It is possible that HPF1 binding may facilitate de-protonation of Ser residues, improving their nucleophile properties (as at physiological pH the hydroxyl group of Ser is present in its protonated form, being a weak nucleophile) (52). For example, Ser OH-group can be de-protonated by a specifically located His residue, as it takes place in the active sites of serine proteases (52).

#### YB-1

Several attempts to establish a mechanism of PARP1 regulation were made for Y-box-binding protein 1 (YB-1) (23–27), that was found to functionally interact with PARP1 in 2015. Similar to Sam68, YB-1 is an RNA-binding protein that can also interact with single- and double-stranded DNA (107). Similar to p53, YB-1 contains a long, disordered, positively charged CTD in its structure and is prone to multimerization during nucleic acid binding. As a multimer, YB-1 can effectively compete with other DNA-binding proteins despite its weak affinity for DNA as an isolated protein (108) and can facilitate assembly of supramolecular structures containing nucleic acids (108–110). YB-1 is able to physically interact with PARP1 in the absence (24) or presence (26) of damaged DNA. The multifunctional CTD of YB-1 (129–324 amino acid residues) or its proximal part (129–219 amino acid residues) is necessary for this interaction. PARP1 interacts with YB-1 by its DNA-binding domain and other undetermined regions (24). YB-1 is a target for covalent poly(ADP-ribosyl)ation by PARP1 (23). Present in excess, YB-1 is a preferable PAR acceptor (26). In this respect, it can increase overall poly(ADP-ribosyl)ation by directing the reaction towards trans-modification and slowdown of PARP1 auto-modification (26). Functional interactions of YB-1 and PARP1 within the ‘ternary' complex, YB-1-PARP1-DNA, are strictly regulated by stoichiometry. At a [YB-1]:[DNA] ratio of more than 10:1, YB-1 strongly inhibits PARP1 activity by competing with the enzyme for DNA binding (23,24) that results in decreased poly(ADP-ribosyl)ation of both YB-1 and PARP1 (23,24). Poly(ADP-ribosyl)ation of YB-1 eliminates its DNA-binding activity (23). Therefore, when YB-1 is PARylated effectively, it never inhibits PARP1. Moreover, YB-1 interacts with PAR non-covalently, and PAR outcompetes YB-1 binding to DNA (24,25). Non-covalent binding of positively charged YB-1 to PAR polymers during auto-modification of PARP1 stabilizes the catalytically active PARP1-DNA complex and stimulates PAR elongation. This stimulation is observed in the absence of Mg^2+^ ions (26). Based on non-covalent interactions with poly(ADP-ribose) chains, YB-1 inhibits PARG activity, thus prolonging the life span of PAR (24). Overall, YB-1 can regulate the reaction of PAR synthesis throughout: as a DNA-binding protein, PARP1-binding protein, PAR-binding protein and as a highly positively charged protein (26).

## CONCLUSIONS AND PERSPECTIVES

The recent advances in the study of poly(ADP-ribosyl)ation are mainly concerned with the biological role and mode of operation of proteins belonging to the PARP family as well as with different functions of poly(ADP-ribosyl)ation in cells. The key role of this process in mammalian cells has stimulated the publication of excellent reviews (111–113) and journal issues [Mol Aspects Med. 2013; 34(6)] devoted to PARP and poly(ADP-ribosyl)ation as well as to PARP inhibitors. At the same time, the molecular mechanism of this process, which is important for understanding the part played by mono- and poly(ADP-ribosyl)ation in regulation of replication, transcription, DNA repair and protein stability/degradation remains unclear to a large extent. All these events have to be regulated by protein-DNA and protein-protein interactions as well as by interactions of proteins with poly(ADP-ribose). It is known that the function of RNA-binding proteins is also dependent on PARP and poly(ADP-ribosyl)ation (27,114).

The combined action of PARP1 and PARP2 seems important in the regulation of poly(ADP-ribosyl)ation of proteins (43,115) and recently discovered poly(ADP-ribosyl)ation of damaged DNA (32,33,116,117). PARP3 along with other members of the PARP family catalyzing mono(ADP-ribosyl)ation may contribute to the initiation of PAR synthesis under specific conditions (117). It is very likely that the synthesis of short or long and branched PAR polymers may perform different functions during the regulation of cellular processes. The synthesis of branched PAR chains could mainly serve for the creation of non-membranous cell compartments by PAR-induced liquid demixing events (84) necessary for regulation of chromatin remodeling and the subsequent multienzyme processes of DNA repair and transcription. In this regard, the combined action of mono-, oligo- and poly(ADP-ribosyl)-transferases in the cell may ensure multilevel regulation of DNA and RNA metabolism.

Since damaged DNA is not the sole activator of PARP1 (85), it can be assumed that more complicated mechanisms are necessary for well-tuned activation and accuracy of ADP-ribosylation process. Indeed, recent studies have revealed a number of proteins that can not only stimulate or inhibit PARP1 catalytic activity, but also appear to regulate target and PAR length. As PARP inhibition hold great promise in cancer therapy, elucidation of nuances of PARylation mechanism as well as molecular mechanisms of activation and regulation of PAR synthesis in cells may provide a basis for the rational development of new treatment strategies.

The aim of this review was to summarize the current knowledge on the molecular mechanism of poly(ADP-ribosyl)ation catalyzed with PARP1 and its regulation to move further forward our study of this key process in mammalian cells.
